# TENet: Attention-Frequency Edge-Enhanced 3D Texture Enhancement Network

**DOI:** 10.3390/s25030715

**Published:** 2025-01-24

**Authors:** Ying Wang, Tao Fu, Yu Zhou, Qinglei Kong, Wenshuai Yu, Jian Liu, Yi Wang, Bo Chen

**Affiliations:** 1 School of Aerospace, Harbin Institute of Technology Shenzhen, Shenzhen 518055, China; 22s061010@stu.hit.edu.cn (Y.W.); 22s061012@stu.hit.edu.cn (T.F.); kongqinglei@hit.edu.cn (Q.K.); wangyi2021@hit.edu.cn (Y.W.); 2 Key Laboratory of Aerospace RS Big-Data Intelligent Processing and Application of Guangdong Higher Education Institutes, Harbin Institute of Technology Shenzhen, Shenzhen 518055, China; 3 Xi’an Institute of Surveying and Mapping, Xian 710054, China; hb48_zy@163.com; 4 The College of Civil and Transportation Engineering, Shenzhen University, Shenzhen 518060, China; ywsh@szu.edu.cn

**Keywords:** 3D surface texture enhancement, oblique photogrammetry, self-attention mechanism, frequency domain enhancement

## Abstract

Oblique photogrammetry imagery often suffers from uneven resolution and blurred details, leading to poor surface texture quality in 3D reconstructions, particularly for building facades. To address these challenges, we propose a novel Attention-Frequency Edge-Enhanced 3D Texture Enhancement Network (TENet) and introduce a comprehensive 3D texture enhancement pipeline. This pipeline applies 2D texture super-resolution techniques to 3D models for fine-grained texture restoration, enhancing the surface texture quality. TENet leverages attention mechanisms and frequency-domain techniques to improve texture sharpness and edge accuracy. Our approach includes a Region-Resolution Adaptive Enhancement Module (RAEM) and a Frequency-Domain Edge Enhancement Mechanism (FDEEM) to enhance local details and restore critical edge features. The experimental results demonstrate that TENet outperforms existing methods, significantly improving texture quality and 3D reconstruction performance. Ablation studies confirmed the effectiveness of each component in enhancing 3D texture reconstruction. The network is validated for real-world applications, showing its ability to significantly reduce edge artifacts and restore clear, accurate textures in real-world 3D surface models.

## 1. Introduction

With ongoing progress in urban environment sensing and analysis in smart cities, along with increasing demands for automation, there has been a significant rise in the need for high-fidelity textured surfaces in 3D modeling based on oblique photogrammetry [1,2]. However, limitations in observation angles, challenges associated with high-angle photography, and variations in lighting conditions often result in reconstructed 3D models with blurry textures and lost details. Furthermore, the performance constraints of current imaging devices exacerbate the degradation of texture quality. Addressing these challenges requires the development of efficient and cost-effective computer vision techniques to enhance the quality of 3D surface textures in oblique photogrammetry [3,4,5].

Existing methods for enhancing 3D surfaces often rely on direct 3D upsampling techniques to refine surface details, such as voxel-based methods [6], point cloud upsampling [7,8,9], mesh subdivision [10], interpolation methods [11], and learning-based upsampling approaches [12,13,14]. While these methods primarily optimize the geometric data of 3D meshes, they fall short in improving texture quality. Additionally, learning-based approaches often encounter difficulties because of the insufficient availability of training data. To overcome these limitations, some studies have explored the use of 2D image Super-Resolution (SR) algorithms as an indirect means to enhance 3D surface quality. For example, Xie et al. developed a multimodal deep neural network for 3D surface super-resolution, operating in the 2D normal domain by integrating texture, depth, and normal information to improve detail recovery and geometric fidelity [15]. Despite achieving enhanced details and geometric precision in theory, the complexity and computational cost of multimodal processing limit its practical applicability. Ranade et al. introduced a novel rendering loss-based approach for 3D texture super-resolution that eliminates the need for geometric information during inference and provides a new 3D model dataset [16]. However, their method requires computationally expensive rendering loss calculations and additional datasets, further increasing the overall complexity. Other studies have employed multi-source texture replacement methods, such as He et al., who introduced a method combining UAV remote sensing and ground imagery for mapping textures onto CityGML building models [11]. By extracting textures from various perspectives, viewpoints, and filtering occlusions using deep convolutional networks, this method successfully mitigated texture blurring and distortion, but at the cost of added complexity and increased application costs due to the reliance on additional data acquisition.

Most existing 3D surface enhancement techniques are model-specific and difficult to generalize to complex, large-scale urban scenarios. Furthermore, these methods impose high computational demands for rendering, limiting their practical scalability [17,18,19]. In response to these challenges, this research presents a straightforward and effective approach for texture enhancement using Image Super-Resolution (SISR) [20]. The method involves extracting 2D texture images from 3D models, applying SISR techniques to enhance the texture images, and then mapping the high-resolution textures back onto the 3D model to improve the surface quality. SISR techniques are fundamental to this process, aiming to enhance the resolution of images from low-quality inputs [21]. Early methods in SISR primarily focused on improving the Peak Signal-to-Noise Ratio (PSNR). For example, Dong et al. proposed SRCNN, a convolutional neural network consisting of three layers, designed to convert low-resolution images into high-resolution ones [22]. Later, Ledig et al. introduced SRResNet by incorporating Residual Networks (ResNet) [23]. However, PSNR-oriented methods often produce overly smooth images with insufficient detail. To address this limitation, Johnson et al. proposed perceptual loss to improve visual quality [24]. In SRGAN, Ledig et al. introduced adversarial loss to improve performance, employing Generative Adversarial Networks (GANs) to create high-resolution images with more realistic details. Although GAN-based methods achieved high-fidelity results, they often suffered from geometric distortions, particularly in edge and fine-texture regions [25]. To mitigate such distortions, gradient space constraints can be introduced to provide additional supervision, reducing geometric artifacts [26,27].

In the evaluation of neural network models, particularly for SISR, key performance metrics include the Peak Signal-to-Noise Ratio (PSNR) and Structural Similarity Index (SSIM) [28]. The PSNR quantifies the fidelity between the generated and ground truth images, with higher values indicating better reconstruction quality. However, PSNR primarily measures global image quality and does not fully capture perceptual differences, especially in complex structures and fine textures. SSIM, on the other hand, assesses perceptual similarity by considering luminance, contrast, and structural integrity, offering a more holistic evaluation of image quality. Higher SSIM values indicate greater similarity to the original image and better perceptual quality, especially for complex and detailed regions of the image. Both metrics are essential for understanding the balance between quantitative fidelity and perceptual realism in image reconstruction tasks. In addition to these metrics, visual comparisons play a crucial role in evaluating super-resolution models, as they provide qualitative insights into the preservation of fine details and edge accuracy, which metrics like PSNR and SSIM may not fully reveal. Visual assessments allow for the identification of artifacts, texture preservation, and clarity in regions with intricate structures, further complementing the quantitative metrics.

To overcome these challenges, this study introduces TENet, a super-resolution network for 3D texture enhancement based on self-attention and frequency-domain edge refinement. Specifically designed for improving the textures of 3D building surfaces, the proposed method first extracts 2D texture images from the 3D model and applies SISR techniques to enhance their resolution. The enhanced textures are then mapped back onto the 3D model to achieve refined texture restoration. By leveraging adaptive processing through self-attention mechanisms and high-frequency enhancement in the frequency domain, the method effectively addresses issues such as non-uniform resolution, detail blurring, and high-frequency information loss.

The key contributions of this research are as follows:

- Proposed TENet Architecture: TENet combines a Dynamic Layered Self-Attention Mechanism (DLSA), Gated Edge Attention Module (GEAM), Region-Resolution Adaptive Enhancement Module (RAEM), and Frequency Domain Edge Enhancement Mechanism (FDEEM). These components collectively address the challenges of detail recovery across regions with varying resolutions while enhancing edge fidelity and overall image quality;
- Region-Resolution Adaptive Enhancement Module (RAEM): RAEM dynamically adjusts convolution kernel sizes to handle uneven resolution in oblique photogrammetry, enhancing local details in high-resolution areas and preserving global structure in low-resolution regions, significantly improving 3D building surface texture restoration;
- Frequency Domain Edge Enhancement Mechanism (FDEEM): The FDEEM extracts and enhances high-frequency components in the frequency domain, effectively compensating for the limitations of spatial convolutions in recovering high-frequency details. This ensures the preservation of fine textures on building surfaces.

## 2. Related Works

This study focuses on addressing the challenges of texture super-resolution for 3D surfaces by leveraging advancements in single-image super-resolution (SISR) techniques, along with custom enhancements for 3D surface textures. This section reviews the development and implementation of SISR methods, as well as their integration into 3D surface texture enhancement, highlighting the limitations of existing approaches and the motivation for the proposed methodology.

### 2.1. Single-Image Super-Resolution (SISR)

Single-Image Super-Resolution (SISR) has evolved significantly, shifting from traditional interpolation techniques to more advanced deep-learning-based methods. Earlier methods, such as bilinear and bicubic interpolation, relied on simple mathematical models, but these methods often introduced blurring and artifacts, particularly in high-frequency regions, thereby limiting the quality of reconstructed images. Subsequently, sparse representation-based approaches were introduced, which involve the construction of sparse dictionary mappings between Low-Resolution (LR) and High-Resolution (HR) images. These methods significantly improved the preservation of fine details. In more recent years, deep learning techniques have gained considerable attention, with SRCNN playing a pioneering role by employing end-to-end convolutional neural networks to directly learn the mapping between LR and HR images, resulting in significant performance improvements [22]. Following this, Enhanced Deep Residual Networks (EDSRs) utilized deeper architectures and skip connections to further improve image sharpness and detail restoration [29]. Generative Adversarial Networks (GANs), such as SRGAN, introduced perceptual loss functions to focus not only on pixel-level reconstruction, but also on capturing high-frequency details, producing images that are more perceptually realistic [23].

Although progress has been made, SISR continues to encounter difficulties in real-world applications. For example, texture misalignment and detail distortion often occur, particularly when processing straight textures on building surfaces, leading to unnatural distortions or blurring at edges, which degrade overall image fidelity. Moreover, most existing SISR datasets assume uniform resolution across the image. However, in practical scenarios, such as UAV imagery, images often exhibit non-uniform resolution: regions closer to the camera have higher resolution, while distant regions suffer from lower resolution. Traditional SISR methods are not well-suited to handle such variation, often resulting in inconsistent quality across regions and ultimately impacting the overall visual appearance. Addressing the challenges of non-uniform resolution, maintaining consistency, and preserving detail fidelity across varying resolution regions remains an important focus of current research.

SISR enhances the resolution of 2D images by learning the mapping between low-resolution (LR) and high-resolution (HR) images to restore details. Despite improvements in sparse representation and deep learning, these methods often assume uniform resolution and overlook variations within the image. Moreover, 2D texture enhancement only deals with pixel-level details in a flat plane, without considering the interaction between texture and geometric structure, limiting its application in 3D scenarios. In contrast, 3D surface texture enhancement must address the complex interaction between texture and geometry, with the goal of not only improving texture details, but also ensuring alignment and consistency with the underlying 3D structure.

### 2.2. Super-Resolution for 3D Surface Textures

In the field of 3D surface texture super-resolution, various methods have been explored to enhance detail recovery and geometric fidelity by integrating different types of data. Xie et al. proposed a method that combines texture, depth, and normal information within the 2D normal domain to enhance 3D surface details [15]. While this approach demonstrated significant improvements, its complexity and high computational cost have limited its widespread applicability. Ranade et al. introduced a rendering loss method to reduce reliance on geometric information and proposed a new 3D model dataset [16]. However, the approach remains computationally intensive and requires additional data inputs, which are challenging in large-scale applications. Li et al. modified the EDSR architecture to incorporate texture maps and normal maps [30]. Although effective, the generation of normal maps introduces significant computational overhead, particularly when dealing with a large number of frames. Similarly, Richard et al. integrated redundancy-based and prior-based methods into a single super-resolution network to create new texture maps [31]. While their approach advanced texture recovery, the high computational demands of rendering and integrating geometric information limited its scalability.

Current approaches in 3D surface texture super-resolution have achieved significant advancements, but most suffer from high computational complexity, particularly when integrating rendering, normal, and geometric information. To address these challenges, this study proposes a decoupled approach that separates texture mapping from surface enhancement. Specifically, texture mapping is first performed to extract 2D texture images, which are then processed using single-image super-resolution techniques. By decoupling these processes, the proposed method effectively reduces overall computational complexity while preserving critical texture details. The experimental results indicate that this method enhances texture quality while considerably reducing computational costs, thereby making it more suitable for complex 3D surface super-resolution tasks in real-world applications.

## 3. Materials and Methods

**Figure 1:**
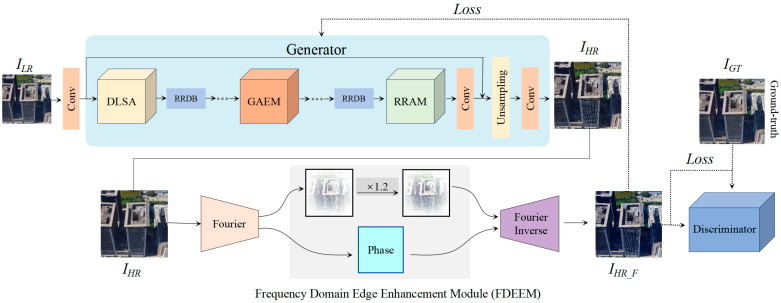
Diagram of TENet network architecture.

The proposed 3D texture enhancement method addresses the challenges of oblique photogrammetry, where input images often exhibit uneven resolution, leading to difficulties in achieving both global consistency and local detail recovery. As shown in Figure 1, the TENet network architecture consists of four key modules: (1) Dynamic Layered Self-Attention (DLSA), which combines local high-resolution details with global structural features to ensure consistency; (2) Gated Edge Attention Module (GEAM), which enhances edge details through multi-scale convolution and gating mechanisms; (3) Region-Resolution Adaptive Enhancement Module (RAEM), which adjusts convolution operations dynamically to recover details across varying resolution regions; and (4) Frequency-Domain Edge Enhancement Mechanism (FDEEM), which strengthens high-frequency features in the frequency domain before reconstructing the final output.

In terms of loss calculation, the TENet network employs two main loss functions. First, the generator’s loss function consists of perceptual loss, pixel-level L1 loss, and adversarial loss. The perceptual loss optimizes the consistency between the generated image, I_HR_F_, and the ground truth image at high-level features. The L1 loss measures the pixel-wise differences, while the adversarial loss, through the Generative Adversarial Network, improves the visual realism of the image. The generator and discriminator collaborate through an adversarial process. The goal of the generator is to produce images, I_HR_F_, that are as realistic as possible, while the discriminator provides feedback by distinguishing between the generated image and the real image. The discriminator’s loss function measures its ability to differentiate between real and generated images, and the generator adjusts its output through the adversarial loss, pushing the generated image closer to the real image.

**Figure 2:**
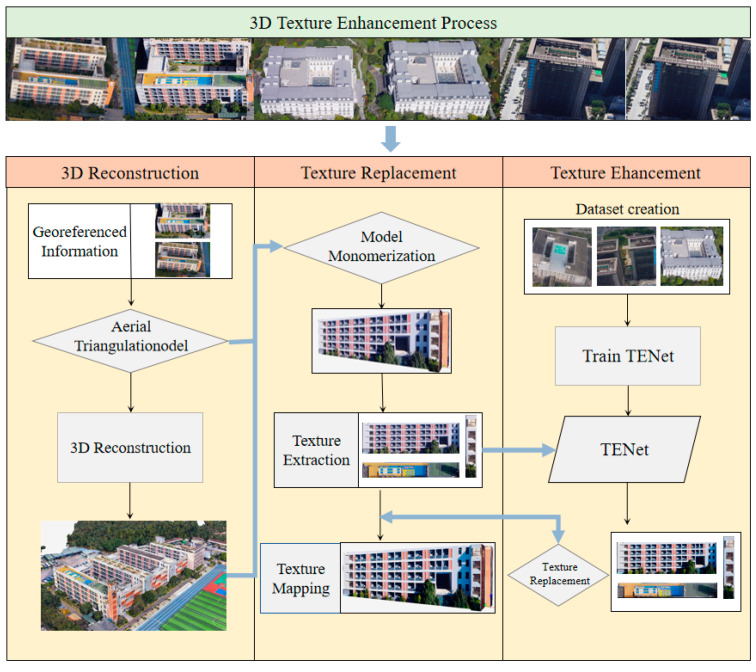
Workflow of 3D texture enhancement process.

The integration of TENet into the overall 3D texture enhancement workflow is depicted in Figure 2. The process begins with the 3D reconstruction stage, where georeferenced information and oblique imagery are used to perform aerial triangulation and generate a textured 3D model. In the subsequent texture replacement stage, 2D texture images are extracted from the model through monomerization. In the final texture enhancement stage, the extracted textures are enhanced using TENet, which applies super-resolution techniques to improve texture clarity and resolution. The enhanced textures are then re-mapped onto the 3D model, replacing the original textures to produce a refined 3D model.

By combining the 3D reconstruction workflow with TENet’s texture enhancement capabilities, the method effectively addresses issues such as texture blurriness and resolution inconsistencies, resulting in high-quality 3D models with improved detail and visual fidelity.

### 3.1. Dynamic Layered Self-Attention Mechanism, DLSA

In the task of enhancing 3D surfaces generated from oblique photogrammetry, input images often exhibit uneven resolution, where different regions of the same image have significantly varying levels of detail. Traditional convolutional operations struggle to handle these inconsistencies effectively, often leading to insufficient local detail recovery or distorted global structures. To address this issue, this study proposes the Dynamic Layered Self-Attention Mechanism (DLSA), an efficient attention-based structure specifically designed to extract multi-resolution features.

**Figure 3:**
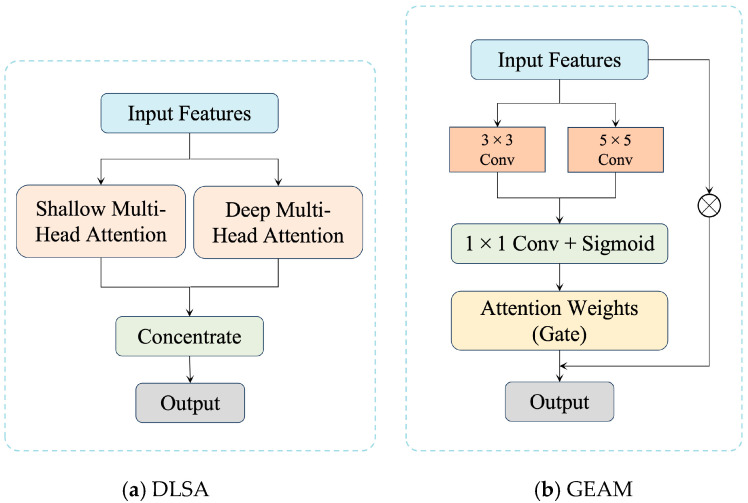
Design of DLSA module and GEAM in TENet.

The core concept of DLSA is to decompose the input feature map into high-resolution and low-resolution regions, applying independent self-attention mechanisms to each. This enables the network to efficiently capture both local details and global structures while maintaining accuracy. Figure 3a illustrates the structure of the DLSA module, where shallow and deep self-attention modules operate in parallel. The Shallow Multi-Head Self-Attention Module is designed to process high-resolution regions, focusing on capturing short-range dependencies such as edges and textures, ensuring accurate detail restoration. Conversely, the Deep Multi-Head Self-Attention Module handles low-resolution regions, capturing long-range dependencies to reconstruct global structural relationships.

In implementation, suppose the input feature map has dimensions. The DLSA module initially divides the feature map into high-resolution and low-resolution regions. In each self-attention mechanism, the feature matrix is processed using the multi-head self-attention (MHSA) operation. The attention calculation is expressed as follows:

(1) $$\mathrm{Att}(Q,K,V)=\mathrm{softmax}\left(\frac{QK^{T}}{\sqrt{d_{k}}}\right)V$$

where Q(Query), K(Key), and V(Value) represent the query, key, and value matrices derived from linear transformations of the input features, with the dimensionality of the key vectors denoted as $d_{k}$. High-resolution features are processed by the shallow self-attention module to capture fine details, while low-resolution features are processed by the deep self-attention module to strengthen global feature representation.

Finally, the outputs from the two modules are concatenated along the channel dimension and passed to subsequent network layers. By dynamically adapting to regions of varying resolution, the DLSA module effectively ensures both local detail recovery and global structural consistency, addressing a critical challenge in texture enhancement for oblique photogrammetry.

### 3.2. Gated Edge Attention Module, GEAM

To enhance key edge features, such as straight lines and fine structures at corners, in the task of super-resolution for 3D building surfaces, the Gated Edge Attention Module (GEAM) adopts a gated mechanism and multi-scale convolutional operations. These techniques effectively extract and dynamically strengthen the salient edge features in the image. Traditional super-resolution methods often struggle with blurred edges or loss of details when dealing with complex building structures. GEAM addresses these challenges through the following two steps.

#### 3.2.1. Multi-Scale Edge Feature Extraction

In the task of super-resolution for 3D building surfaces, edge features of varying scales and orientations are critical for enhancing geometric details such as lines and corners. To precisely extract these edge features, the Gated Edge Attention Module (GEAM) employs multi-scale convolution operations to capture fine edges from multiple perspectives.

As shown in Figure 3b, the GEAM module employs 3 × 3 and 5 × 5 convolutional kernels to capture edge features at various scales and orientations. This multi-scale convolution design ensures that edge details in horizontal, vertical, and diagonal directions are effectively captured. For instance, horizontal convolutional kernels enhance the clarity of straight features like window frames and rooftops, while vertical convolutional kernels focus on identifying edges, such as the junctions between walls and the ground. Furthermore, the module incorporates convolutional kernels oriented for diagonal and curved structures to handle more complex geometric patterns, ensuring comprehensive edge extraction from all directions.

Mathematically, assuming the input image is I, the edge features extracted through multi-scale convolution operations can be expressed as:

(2) $$E_{i}=\sum_{i}I*W_{K_{i}}$$

where $E_{i}$ represents the edge features extracted at the i-th scale, $K_{i}$ denotes the convolutional kernel of the i-th scale and orientation, and ∗ indicates the convolution operation. By using convolutional kernels of different scales, GEAM captures edge information from multiple perspectives, effectively avoiding the edge blurring issues commonly encountered in traditional super-resolution methods.

#### 3.2.2. Dynamic Saliency Assessment and Weight Assignment

To further refine edge features, the GEAM module introduces a gating mechanism (SA-Gate) to dynamically assign enhancement weights based on edge saliency. By leveraging attention mechanisms, GEAM identifies critical edges essential for building structures and assigns higher weights to these salient features, ensuring that these features are emphasized in the final high-resolution image.

Initially, GEAM evaluates the saliency of each pixel in the edge features using an attention mechanism, producing a saliency weight matrix *A*, which reflects the relative importance of each pixel. For critical edges, such as window frames or the junctions between walls and ground, the saliency scores are higher, resulting in larger enhancement weights. In contrast, less important or noisy edges are suppressed to avoid artifacts caused by over-enhancement.

The operation can be expressed as:

(3) $$E_{\mathrm{enhanced}\;}=A⊙E$$

The enhanced edge features are represented by $E_{enhanced}$, with *A* denoting the saliency weight matrix. E refers to the extracted edge features, and ⊙ represents element-wise multiplication operator. This mechanism ensures that key edges are enhanced while noisy edges are suppressed, maintaining the clarity and naturalness of the generated high-resolution image.

By combining multi-scale edge extraction and dynamic saliency assessment, the GEAM module not only captures edge features of varying scales, but also dynamically enhances critical edges while suppressing irrelevant ones. This approach ensures that important structural details, such as straight lines and corners, are preserved in the super-resolved images. In comparison with conventional super-resolution techniques, GEAM effectively avoids edge blurring and artifacts, significantly improving the geometric accuracy and detail restoration of 3D building surfaces.

### 3.3. Region-Resolution Adaptive Enhancement Module, RAEM

Images captured through oblique photogrammetry often exhibit uneven resolution. Specifically, regions closer to the camera typically have higher resolution and provide clearer details, whereas distant regions have lower resolution due to the greater camera-to-object distance. This resolution disparity poses significant challenges for traditional super-resolution networks, as uniform convolution kernels cannot simultaneously adapt to the detailed feature extraction required in high-resolution regions and the global structure recovery needed in low-resolution regions. To overcome this challenge, we introduce the RAEM, which dynamically adjusts convolution kernel sizes based on the resolution variations of the input image. This mechanism ensures consistency in both local detail recovery and global structural coherence by adapting the receptive field to match the resolution characteristics of different regions.

#### 3.3.1. Resolution Awareness and Detection

**Figure 4:**
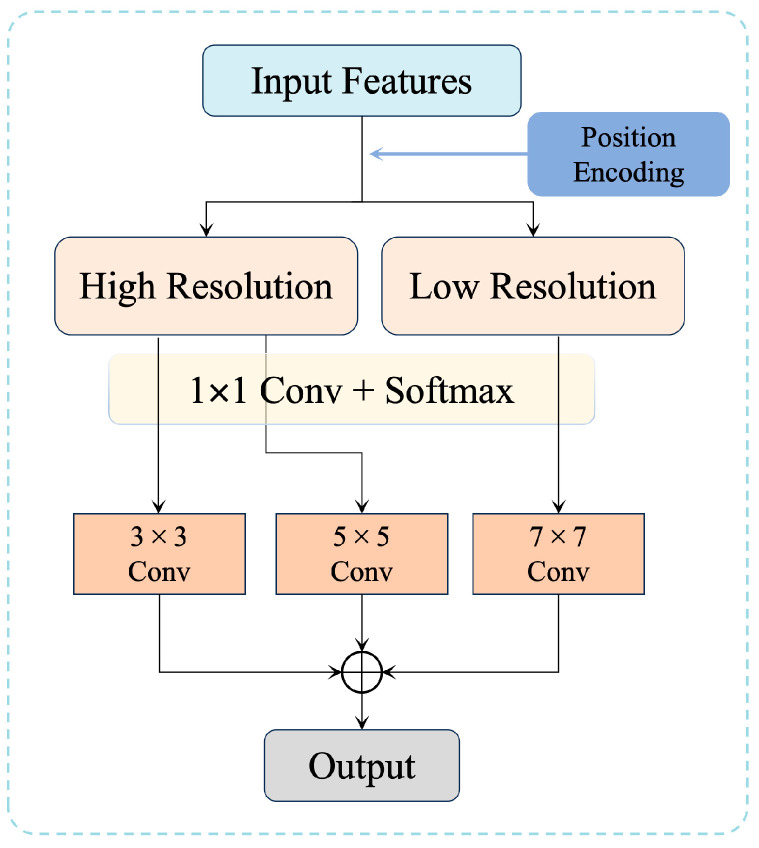
Design of the Region-Resolution Adaptive Enhancement Module (RAEM).

The resolution awareness mechanism in RAEM (highlighted in blue in Figure 4) combines position encoding with local frequency feature analysis. This integration enables the automatic identification of high-resolution and low-resolution regions, guiding adaptive convolution operations in subsequent stages.

1. Position Encoding. Position encoding is a key component of the resolution awareness mechanism. It embeds spatial position information into the feature map by encoding the coordinates of each pixel, allowing the network to identify resolution variations within the image. The position encoding formula is as follows:
  (4) $$P(x,y)=\left\{\begin{array}{ll} \sin\left(\frac{y}{10000^{2i/d}}\right), & \;\mathrm{if}\;i\;\mathrm{is}\;\mathrm{even}\;\\ \cos\left(\frac{x}{10000^{2i/d}}\right), & \;\mathrm{if}\;i\;\mathrm{is}\;\mathrm{odd}\; \end{array}\right.$$
  where *x*, *y* are the spatial coordinates of the pixel, *C* represents the feature dimension, and *k* is the index corresponding to the feature dimension. This encoding scheme integrates pixel position with the information in the feature map, enabling the network to differentiate between high-resolution and low-resolution regions.
2. Local Frequency Feature Analysis. To further distinguish resolution characteristics, the resolution awareness mechanism analyzes local frequency features within the image. High-frequency regions typically correspond to high-resolution details, such as edges and textures, while low-frequency regions are associated with flat areas or background structures. Local frequency features are computed using the Fourier transform, as illustrated below:
  (5) $$F(u,v)=\sum_{x=0}^{M-1}\sum_{y=0}^{N-1}I(x,y)e^{-j2\pi \left(\frac{ux}{M}+\frac{vy}{N}\right)}$$
  In this equation, *I*(*x*,*y*) denotes the pixel intensity in the spatial domain and *F*(*u*,*v*) is the frequency domain representation. *M* and *N* are the image dimensions, and *u*,*v* are the frequency domain coordinates.

By combining position encoding and frequency analysis, the resolution awareness mechanism outputs the regional resolution features *R*, which can be represented as:

(6) R=f(PE,F)

*PE* refers to the position encoding features and *F* denotes the local frequency features.

#### 3.3.2. Adaptive Convolution Kernel Adjustment

Once the resolution awareness mechanism identifies regions of differing resolution, RAEM dynamically adjusts the convolution kernel sizes to process each region appropriately.

1. High-Resolution Regions. High-resolution areas often contain detailed edges and textures. RAEM assigns smaller convolution kernels (3 × 3) to these regions to ensure precise feature extraction, minimizing detail blurring.
2. Low-Resolution Regions. Low-resolution areas typically emphasize global structural recovery. Larger convolution kernels (e.g., 5 × 5 or 7 × 7) are applied to capture broader contextual information, ensuring global coherence.

The adaptive convolution kernel adjustment can be represented as:

(7) $$O=H\left(K_{s}*I\right)+L\left(K_{l}*I\right)$$

In this equation, *O* denotes the output feature map, *K*_*s*_ and *K*_*l*_ represent the small and large convolution kernels. *H* and *L* are indicator functions for the high-resolution and low-resolution regions, respectively, and ∗ denotes the convolution operation.

#### 3.3.3. Dynamic Weight Allocation and Convolution Operations

In addition to adaptive convolution kernel adjustment, RAEM assigns dynamic processing weights to regions with varying resolution. High-resolution regions are assigned higher enhancement weights to prioritize critical details, while low-resolution regions are assigned moderate weights to maintain global consistency. The weight allocation process is guided by the output of the resolution awareness mechanism and is expressed as:

(8) O=W⊙I

*O* is the final output feature map, *I* is the input, and *W* is the dynamically assigned weight matrix. The ⊙ operator denotes element-wise multiplication. This mechanism ensures that important details in high-resolution regions are effectively enhanced, while irrelevant information is suppressed.

By integrating resolution awareness, adaptive convolution kernel adjustment, and dynamic weight allocation, RAEM effectively handles the resolution disparity in oblique photogrammetry images. It ensures that critical details are preserved in high-resolution regions and that global structure is accurately recovered in low-resolution regions. Compared with traditional super-resolution methods, RAEM significantly improves both the local detail restoration and global structural consistency of 3D building surfaces.

### 3.4. Frequency Domain Edge Enhancement Mechanism, FDEEM

**Figure 5:**
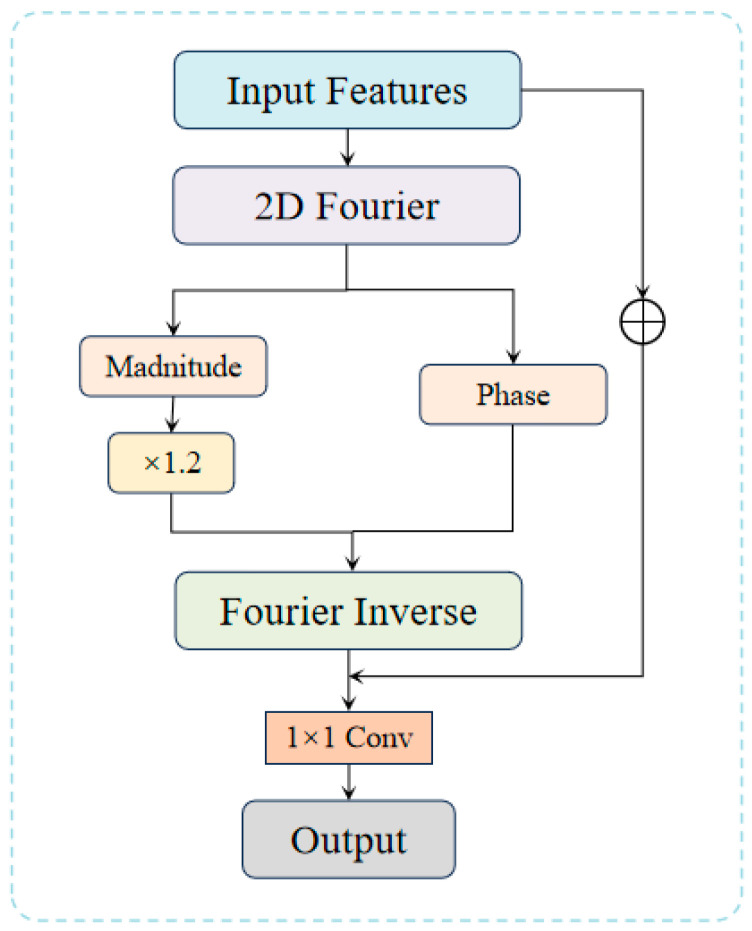
Design of the Frequency Domain Edge Enhancement Module (FDEEM).

Although the Gated Edge Attention Module (GEAM) enhances significant edge details in the spatial domain through a gating mechanism and effectively suppresses unnecessary edges and noise, convolution operations in the spatial domain inherently have limitations in fully recovering high-frequency information. This limitation is especially noticeable in maintaining fine high-frequency details, such as window frames and wall edges. To address this issue, this study introduces the Frequency Domain Edge Enhancement Mechanism (FDEEM), which leverages frequency domain processing to further enhance the high-frequency components of an image, thereby improving edge clarity and detail fidelity on building surfaces, as illustrated in Figure 5.

#### 3.4.1. High-Frequency Component Extraction and Enhancement

The core of FDEEM lies in extracting and enhancing high-frequency information. High-frequency components in an image are associated with edges and textures, whereas low-frequency components correspond to smoother regions. As discussed in the RRAM module, frequency features can be separated using Fourier transformation. Building on this, FDEEM extracts and enhances the image’s high-frequency features using an adaptive weighting factor. To prevent the loss of high-frequency details, FDEEM amplifies these regions through a targeted enhancement factor, ensuring that edges are prominently preserved in the final output. The process of enhancing high-frequency components can be described as follows:

(9) $$F_{enhanced}\left(u,v\right)=\alpha \cdot F\left(u,v\right)$$

*F*(*u*,*v*) denotes the extracted high-frequency components and *α* is the enhancement factor that controls the intensity of high-frequency amplification.

#### 3.4.2. Fusion of Frequency and Spatial Domains

After enhancing the high-frequency components, FDEEM performs an inverse Fourier transformation to convert the frequency domain information back into the spatial domain. The enhanced high-frequency features are then fused with the previously processed spatial domain features. This fusion ensures that the global structure of the image is preserved while the edge details are accurately recovered. The inverse Fourier transformation can be expressed as:

(10) $$f(x,y)=\frac{1}{MN}\sum_{u=0}^{M-1}\sum_{v=0}^{N-1}F(u,v)\cdot e^{j2\pi \left(\frac{ux}{M}+\frac{vy}{N}\right)}$$

This integration enables FDEEM to improve edge details while preserving global structural consistency, thereby enhancing the visual quality of the super-resolved image.

### 3.5. Data Description

**Figure 6:**
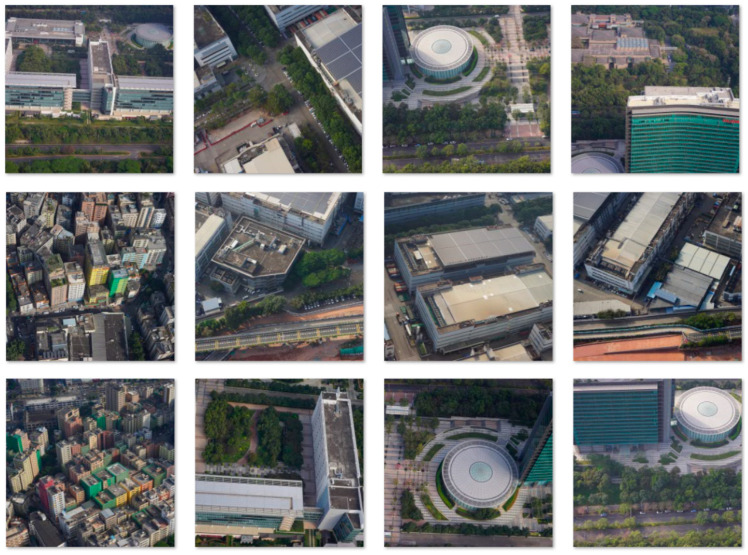
Diagram of the training dataset.

The training dataset used in this study consists of high-resolution oblique photogrammetry images sourced from the original imagery data of drone-based 3D reconstruction. The dataset includes both building and non-building elements, such as grass, roads, and other landscape features. The original resolution of each image sample is 6000 × 4000 pixels. To improve training efficiency and make full use of computational resources, various data augmentation techniques were employed, including random horizontal flipping, rotation up to 30°, affine transformation, and scaling. For compatibility with the model input, all images were cropped to a size of 512 × 512 pixels during preprocessing. Sample images from the training dataset are shown in Figure 6. The entire dataset comprises 454 samples, with 80% used for training and validation, and the remaining 20% used for testing. The experimental test dataset includes four publicly available super-resolution datasets: Set5, Set14, BSD100, and Urban100. The training dataset is non-public, and access to the data and resources can be requested by contacting the authors directly.

### 3.6. Module Analysis and Summary

In this chapter, we proposed four key modules and the dataset design for enhancing 3D surface textures. To better understand the relationships between these modules, we will now discuss the complementary roles of FDEEM and GEAM.

During the edge enhancement process, FDEEM and GEAM work in a complementary manner across different processing domains to improve edge and detail recovery. Specifically, GEAM operates in the spatial domain to perform an initial enhancement of significant edges, while FDEEM refines high-frequency details through frequency do-main processing.

GEAM focuses on selectively enhancing key edges in the spatial domain using attention mechanisms. By targeting prominent edges such as straight lines, corners, and junctions, GEAM achieves precise edge enhancement. Its multi-scale convolution operations effectively reduce noise and suppress irrelevant details, thereby highlighting the structural features of the building. However, despite its effectiveness in processing significant edges, GEAM faces limitations in fully restoring high-frequency details, particularly those involving complex textures and fine edges.

FDEEM overcomes these limitations by shifting the image to the frequency domain, where high-frequency components can be processed directly. By enhancing high-frequency details in the frequency domain, FDEEM compensates for the inherent shortcomings of spatial domain convolutions. This approach enables the module to accurately restore fine edge and texture details, particularly those that are challenging to capture in the spatial domain.

In summary, the proposed TENet integrates self-attention mechanisms with frequency domain processing to address issues such as uneven resolution, edge blurring, and high-frequency detail loss. This combination provides an effective solution for enhancing 3D surface textures in oblique photogrammetry.

## 4. Results

### 4.1. Implementation Details

The models were trained on an NVIDIA GTX 3090 GPU with the Adam optimizer, starting at a learning rate of 0.0002. The learning rate was progressively reduced to near zero using a cosine decay schedule during training. The loss function incorporated L1 loss, perceptual loss, and GAN loss to balance the recovery of both low-frequency structures and high-frequency details.

To enhance computational efficiency and stabilize the training process, a phased freezing strategy was adopted. In the initial 50 epochs, certain model parameters were frozen, while in the following 50 epochs, all parameters were unfrozen for fine-tuning.

### 4.2. Compared with State-of-the-Art Methods

To assess the performance of the proposed TENet, we compared it with existing state-of-the-art methods, such as DLGSANet [32] and CAMixerSR [33]. The evaluation was carried out on widely used benchmark datasets, including Set5, Set14, BSD100, and Urban100. The training dataset used in this study is the proposed oblique photogrammetry dataset, which includes both building structures and non-building elements, such as roads and vegetation. We used PSNR and SSIM as quantitative metrics for comparison, with the results presented in Table 1. Additionally, a qualitative evaluation was performed through visual analysis to further assess the texture enhancement results. Urban100 was selected as the primary dataset for quantitative evaluation, given its closer alignment with the specific characteristics of our research scenario.

**Quantitative Analysis**: TENet demonstrated superior performance across all datasets. On Set5, TENet attained a PSNR of 23.8 and an SSIM of 0.63, outperforming DLGSANet and CAMixerSR by a significant margin. Similarly, on Urban100, where fine details and structural consistency are crucial, TENet achieved a PSNR of 23.4 and an SSIM of 0.61, surpassing the respective metrics of CAMixerSR and DLGSANet. These results validate the robustness of our method in handling diverse and complex image structures.

**Figure 7:**
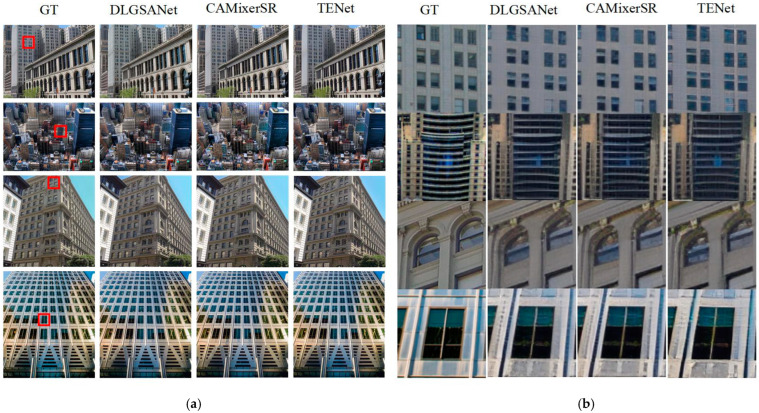
Visual comparison of super-resolution results. (**a**) Ground truth (GT), DLGSANet, CAMixerSR, and TENet outputs for the entire scene; (**b**) Enlarged details of the red box regions showing performance differences in texture reconstruction and edge sharpness.

**Qualitative Analysis**: Figure 7 compares the visual results of TENet with those of DLGSANet and CAMixerSR. TENet consistently restores sharper edges, finer textures, and enhanced geometric fidelity. In urban scenes, where preserving structural regularity is critical, TENet effectively reconstructed straight building edges and window details, which appear distorted or oversmoothed in the outputs of the baseline methods. For instance, in the second row of Figure 7, the building edges reconstructed by TENet are well-aligned and geometrically accurate, whereas CAMixerSR introduces noticeable distortions, and DLGSANet fails to capture the sharpness.

The outstanding performance of TENet arises from its unified framework, which incorporates the Dynamic Layered Self-Attention (DLSA), Gated Edge Attention Module (GEAM), Region-Resolution Adaptive Enhancement Module (RAEM), and Frequency-Domain Edge Enhancement Module (FDEEM). Specifically, DLSA and GEAM effectively enhance edge clarity and multi-scale detail recovery, while RAEM adapts to non-uniform resolution variations and FDEEM refines high-frequency details in the frequency domain. Together, these modules synergistically address the challenges posed by complex textures and varying resolutions.

Overall, both quantitative and visual assessments demonstrate that TENet surpasses existing methods in achieving superior detail preservation and structural consistency, confirming its efficacy in 3D texture enhancement tasks.

### 4.3. Ablation Studies

To evaluate the impact of each module, we conducted experiments using six different model configurations, all trained on a 4× super-resolution task to ensure consistency. The results indicate that the introduction of RAEM and FDEEM significantly improved the network’s performance. The objective evaluation metrics are shown in Table 2.

- **Base Model**: Without any added modules, the base model achieved a PSNR of 22.3256 dB and SSIM of 0.5931, which highlights the model’s limited ability to reconstruct images and recover high-frequency details;
- **DLSA and GEAM**: The addition of the Dynamic Layered Self-Attention (DLSA) mechanism and Gated Edge Attention Module (GEAM) improved the performance, with PSNR increasing to 22.9462 dB and SSIM to 0.6252, improvements of 0.62 dB and 0.03 over the base model, respectively. DLSA enhanced the ability to capture local image details, while GEAM optimized edge clarity and continuity;
- **RAEM**: Introducing RAEM further improved performance, with PSNR increasing to 22.9967 dB and SSIM to 0.6358. Notably, the improvement in SSIM demonstrates that RAEM addressed resolution inconsistencies and enhanced the global feature consistency of the image;
- **FDEEM**: When FDEEM was added alone, PSNR reached 23.0501 dB and SSIM was 0.6148, showing a significant increase in PSNR but a slight decrease in SSIM compared with other combinations of modules. This suggests that the sharpening effect introduced by FDEEM may have overemphasized local edges, causing slight structural distortions and a reduction in the overall structural similarity;
- **DLSA, FDEEM, and GEAM**: Combining DLSA, FDEEM, and GEAM resulted in a PSNR of 22.9967 dB and SSIM of 0.6158. While PSNR improved, the performance did not surpass that of FDEEM alone (PSNR = 23.05, SSIM = 0.61). This could be due to redundancy or conflicts in the features extracted by the modules, limiting their synergistic effect;
- **Full Module Combination**: When all four modules were used together, PSNR increased significantly to 23.9368 dB and SSIM reached 0.6291. This represents an improvement of 1.61 dB and 0.04 over the base model. Compared with the combination of DLSA, FDEEM, and GEAM, the performance improved by 0.94 dB and 0.01. Although the combination of all four modules showed an improvement in PSNR, the SSIM did not surpass that of the standalone RAEM due to feature conflicts between the modules and the sharpening effect introduced by FDEEM. This result highlights the importance of the RAEM module in global optimization and the effective collaboration of all modules to address potential conflicts and redundancy between local features, leading to a more stable and high-quality reconstruction.

**Figure 8:**
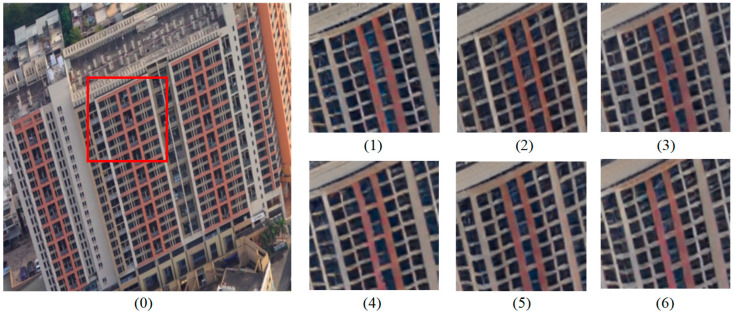
Visual comparison of ablation study. (**0**) Ground truth; (**1**–**6**) correspond to the results of the six different ablation study configurations.

To support the impact of each module, we compared the visual results of the ablation study. In Group 0 (baseline), the texture and edges are blurred, with visible distortions in straight lines. By introducing DLSA + GEAM, local details improve, and the edges become sharper. After adding the RAEM module, the image shows higher consistency with a noticeable resolution improvement. The FDEEM module alone sharpens the image, but overemphasizes edge details, causing slight structural distortions. In Figure 8(5), texture sharpening is too aggressive, and the structure deviates significantly from the ground truth. However, as the modules are combined, the image structure is restored to closely match the ground truth under the influence of RAEM, with clearer straight lines, reduced edge blurring, and fewer edge artifacts, resulting in the best performance.

### 4.4. 3D Texture Mapping Experiment

**Figure 9:**
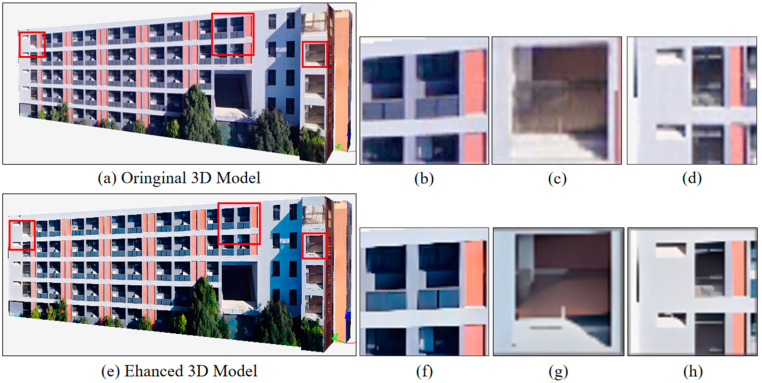
(**a**) Overall view of the original 3D model, directly reconstructed without texture enhancement. (**b**–**d**) Zoomed-in views of specific areas in the original 3D model. These details reveal low texture clarity and noticeable blurriness in fine structures. (**e**) Overall view of the enhanced 3D model, reconstructed with texture enhancement applied. (**f**–**h**) Corresponding zoomed-in views of the enhanced 3D model.

Figure 9 compares the original and enhanced 3D models, highlighting both overall views and zoomed-in details to evaluate the improvements achieved through texture enhancement in the reconstruction process.

The overall view of the original 3D model (Figure 9a) reveals that the model reconstructed directly from oblique imagery, without any texture enhancement, suffers from low texture clarity and insufficient detail representation. In the zoomed-in views (Figure 9b–d), it is evident that critical regions, such as windows, frames, and walls, exhibit significant blurriness. The fine structures, particularly high-frequency elements like window edges and building contours, are poorly represented, with unnatural edge transitions and missing texture details. This highlights the limitations of the original model in scenarios requiring high-resolution and high-fidelity texture representation.

In contrast, the overall view of the enhanced 3D model (Figure 9e) demonstrates a substantial improvement in texture clarity after applying the proposed texture enhancement method. The zoomed-in views (Figure 9f–h) further validate these improvements. Specifically, the window edges (Figure 9f) are sharper and more distinct, the wall’s shading and texture details (Figure 9g) are better defined, and the intersections of building structures (Figure 9h) exhibit precise geometric boundaries and texture fidelity. These results suggest that the TENet framework successfully recovers high-frequency details, enhancing the overall quality of the reconstructed model.

Overall, the comparison demonstrates that the enhanced 3D model not only achieves greater visual clarity, but also preserves structural details with higher accuracy. This confirms the effectiveness of the proposed method in tackling texture blurriness and detail loss in 3D reconstruction tasks.

### 4.5. Discussion

The experimental results demonstrate the excellent performance of the TENet framework in texture enhancement and 3D reconstruction tasks. Compared with existing baseline methods, TENet shows significant improvements in both PSNR and SSIM, particularly in urban scenes, where it effectively reduces edge and texture artifacts, restoring sharp and accurate edges. Its advantage lies in the synergistic operation of multiple modules, including DLSA, GEAM, RAEM, and FDEEM, which enhance edge clarity, address resolution variations, and refine high-frequency details.

However, despite the improvement in PSNR with the combination of all four modules, the SSIM did not surpass that of RAEM alone, indicating that there is still room for the further optimization of the module collaboration, particularly in reducing feature redundancy and improving structural preservation.

The 3D experimental results further validate the effectiveness of TENet. By applying the texture enhancement method, the overall resolution was significantly improved, and the details of the 3D model were better restored, particularly in key areas such as windows and walls, with clearer structures and more realistic textures. This enhancement not only improves visual quality, but also provides a feasible implementation path for engineering applications, demonstrating the potential of TENet for 3D surface texture enhancement in real-world scenarios. Future work could focus on optimizing the network structure and improving network efficiency to further enhance performance and processing speed.

## 5. Conclusions

In this study, we introduced TENet, an innovative attention-frequency edge-enhanced super-resolution network, tailored for enhancing 3D texture details in oblique photogrammetry. The network effectively addresses challenges such as uneven resolution, edge blurriness, and high-frequency detail loss by incorporating four key modules: Dynamic Layered Self-Attention (DLSA), Gated Edge Attention Module (GEAM), Region-Resolution Adaptive Enhancement Module (RAEM), and Frequency Domain Edge Enhancement Mechanism (FDEEM).

The proposed TENet framework outperformed existing state-of-the-art methods, as confirmed by both quantitative metrics (PSNR and SSIM) and visual assessments on several benchmark datasets. The experimental results revealed that the combination of these modules synergistically improves edge clarity, multi-scale detail recovery, and high-frequency texture refinement. In particular, RAEM effectively handles non-uniform resolution variations, while FDEEM enhances fine details in the frequency domain, achieving comprehensive texture enhancement.

Moreover, the application of TENet to 3D reconstruction tasks successfully improved the visual fidelity and structural accuracy of 3D models. The enhanced 3D models displayed sharper edges, more distinct textures, and better alignment with geometric structures, which are critical for real-world urban modeling and visualization applications.

This work not only advances the capabilities of super-resolution techniques for 3D surface texture enhancement, but also sets a new benchmark for integrating spatial and frequency domain processing in computer vision. Future research will aim to enhance the adaptability of TENet to a wider range of datasets and improve its computational efficiency for large-scale urban scenarios.

## Figures and Tables

**Table 1:** Comparison with baseline methods.

Method	Set5		Set14		BSD100		Urban100	
	PSNR	SSIM	PSNR	SSIM	PSNR	SSIM	PSNR	SSIM
DLGSANet	23	0.6	22.5	0.59	22.8	0.61	22.7	0.6
CAMixerSR	23.3	0.61	22.8	0.6	23	0.62	22.9	0.61
ours	23.8	0.63	23.4	0.62	23.3	0.63	23.4	0.61

**Table 2:** Ablation study—impact of different module combinations on PSNR and SSIM.

	Module	Scale	PSNR	SSIM
1	none	×4	22.3256	0.5931
2	dlsa + geam	×4	22.9462	0.6252
3	raem	×4	22.9967	0.6358
4	fdeem	×4	23.0501	0.6148
5	dlsa + fdeem + geam	×4	22.9967	0.6158
6	dlsa + fdeem + geam + raem	×4	23.9368	0.6291

## Data Availability

Data are contained within the article.
